# Thyroid Nodule Experts Evaluating ChatGPT’s Assessment of Thyroid Nodules Classified by the Bethesda System for Reporting Thyroid Cytopathology

**DOI:** 10.1177/19160216251387617

**Published:** 2025-12-01

**Authors:** Alexander Moise, Luiza Tatar, Noa Sela, Sabrina Daniela da Silva, Jasmine Kouz, Michael Tamilia, Michael P. Hier, Veronique-Isabelle Forest, Richard J. Payne

**Affiliations:** 1 Faculty of Medicine and Health Sciences, McGill University, Montreal, QC, Canada; 2 Azrieli Faculty of Medicine, Bar Ilan University, Safed, Israel; 3 Department of Otolaryngology–Head and Neck Surgery, Jewish General Hospital, Montreal, QC, Canada; 4 Département d’Endocrinologie, Hôpital du Sacré-Cœur de Montréal, Université de Montréal, Montréal, QC, Canada; 5 Division of Endocrinology, Department of Medicine, Jewish General Hospital, Montreal, QC, Canada

**Keywords:** ChatGPT, artificial intelligence, Bethesda, thyroid

## Abstract

**Importance:**

ChatGPT has emerged as a medical resource through advanced language processing. Patients with thyroid nodules classified under The Bethesda System for Reporting Thyroid Cytopathology (TBSRTC) may use it to complement discussions with physicians.

**Objective:**

We aimed to determine whether ChatGPT's recommendations on managing thyroid nodules classified by TBSRTC align with those of experienced thyroid specialists.

**Setting/Participants:**

A multidisciplinary panel of 5 thyroid cancer specialists, including otolaryngologists and endocrinologists, from 3 university-affiliated teaching hospitals in Montreal, Canada, evaluated the responses.

**Intervention/Exposure:**

ChatGPT-3.5 was prompted with 4 questions for each of the 6 Bethesda categories regarding the meaning and management of thyroid nodules, generating 24 responses for evaluation.

**Main Outcome/Measures:**

We assessed ChatGPT’s accuracy against the latest American Thyroid Association (ATA) guidelines using a 4-point Likert scale (<50%, 50-74%, 75-89%, >90%). Additionally, specialists rated their comfort or reluctance in recommending ChatGPT as a complementary tool for patient discussions.

**Results:**

Of the 24 ChatGPT-generated responses, 19 (79.2%) demonstrated moderate to good consistency with the ATA guidelines. The mean consistency score was 3.38/4 and median was 3.5. Consensus (IQR ≤ 1) was achieved in 23 out of 24 responses (95.8%), reflecting strong inter-rater reliability. Consistency scores were highest in Bethesda I–III and declined progressively in higher-risk categories, with the lowest mean score observed in Bethesda VI. Similarly, an upward trend in clinician reluctance was observed from Bethesda I through VI, indicating greater caution in recommending ChatGPT responses for patients suspicious for or diagnosed with malignancy (Bethesda V–VI).

**Conclusion and Relevance:**

While ChatGPT’s responses generally align with specialist recommendations, they are not fully reliable. ChatGPT lacks the ability to serve as an independent or accurate source of medical advice for thyroid nodule management. It remains a useful complement for patient discussions, especially in low-risk scenarios, but further improvements are necessary to make it a safe, reliable component of patient care in complex cases.

## Graphical abstract

**Visual Abstract:**
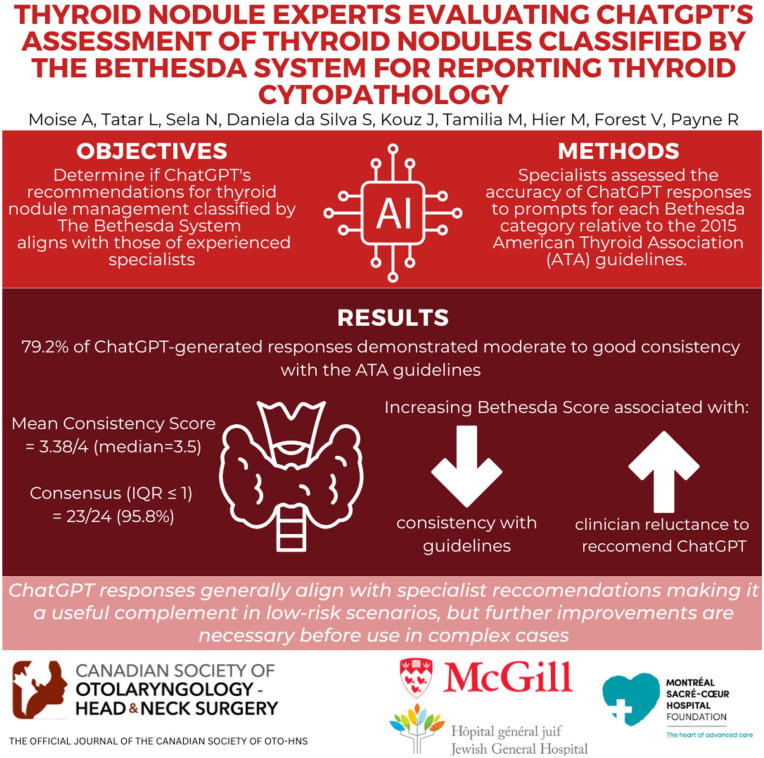

## Key Messages

- ChatGPT’s answers are most to least consistent with current guidelines, including the latest American Thyroid Association Guidelines, for Bethesda categories III, I, II, IV, V, and VI.
- Expert’s comfortability recommending ChatGPT to complement the discussion with the physician decreases the higher the Bethesda Category.
- ChatGPT has potential as a supportive tool in the management of low-scoring Bethesda nodules.

## Introduction

The Bethesda System for Reporting Thyroid Cytopathology (TBSRTC), implemented in 2010 and updated in 2017, has standardized thyroid cytopathological diagnostics.^1^ Based on TBSRTC, thyroid nodules fall in one of six categories corresponding to their rate of malignancy.^1^ This system is critical in making management decisions with patients who have thyroid nodules and is incorporated in the 2015 ATA Guidelines and other similar guidelines widely used by physicians.^2^

Despite containing important diagnostic information, the TBRSTC’s specialized terminology may be difficult to understand for some patients. As a result, many individuals complement their discussion with physicians by using online, publicly available resources, including ChatGPT. OpenAI’s freely available GPT-3.5 is an advanced artificial intelligence (AI) powered language model that excels in generating human-like text responses for a broad spectrum of queries, ranging from basic to complex.^3^ Recent studies have measured its ability to apply the knowledge contained in medical sources across the Internet and showed its capacity to pass the United States Medical Licensing Exam (USMLE) despite lacking formal medical training.^4^ This indicates its potential as a versatile tool for physicians across various medical specialties.^3,5,6^ However, to date, the accuracy of ChatGPT in processing medical data is sub-optimal because it has been found to exhibit biases in selecting academic references and lacks medical nuance in its response.^6,7^ Considering the incertitude related to ChatGPT’s ability at providing accurate information to patients soliciting information about thyroid nodules, this study aimed to compare ChatGPT’s recommendations on the management of thyroid nodules classified under the TBSRTC with those provided by experienced thyroid specialists.

## Methods

To assess the advisory potential of conversational AI regarding the meaning and management options for specific thyroid nodule Bethesda scores, we prompted ChatGPT-3.5 with 4 questions for each nodule category. While patients were not involved in the study design, the questions were developed by thyroid specialists to reflect the type of informational needs and typical inquiries patients commonly express during clinical consultations. A panel of 5 highly specialized thyroid experts—3 otolaryngologists (thyroid surgeons) and 2 endocrinologists from McGill University teaching hospitals in Montreal, Canada—evaluated the accuracy of the AI-generated responses. All panel members are directly involved in thyroid cancer care and the application of clinical guidelines, ensuring that both the question content and evaluation process are grounded in routine clinical practice.

ChatGPT-generated responses were critically evaluated by each specialist against current guidelines (including the ATA 2015 guidelines) using a four-point scale. A score of 1 indicated less than 50% consistency (low); 2 corresponded to 50-74% consistency (low to moderate); 3 reflected 75-89% consistency (moderate to good); and 4 was given for greater than 90% consistency (high). The 4-point scale was intentionally structured with wider intervals at lower-consistency levels and narrower intervals at higher levels to ensure conservative scoring and prevent overestimation of guideline adherence. This approach ensures that only responses demonstrating high certainty and strong adherence to guidelines receive the highest scores. The blinded nature of the assessment precluded evaluators from viewing each other’s ratings, thereby eliminating the potential of peer influence on individual judgments.

In addition, evaluators rated their comfort (1 comfortable; 2 not comfortable) in recommending the use of ChatGPT as a complementary information resource to their patient discussions.

The Research Ethics Office (Institutional Review Board) of the McGill Faculty of Medicine and Health Sciences waived the need for ethical approval according to article 2.5 of the Tri-Council Policy Statement, as this study is a performance review of ChatGPT-3.5 and does not involve patient participation.

### Statistical Analysis

Physician agreement with the ChatGPT-generated responses was analyzed using mean and standard deviation (SD), median and interquartile range (IQR) to represent central tendency and variability. This approach, widely adopted in health research, enhances the robustness of interpretation and offers a more comprehensive view of response patterns and distribution characteristics. The distribution of scores (ie, the number of evaluators assigning scores of 1-4) was also reported to provide transparency. To assess inter-rater agreement, the RAND/UCLA Appropriateness Method was applied, with consensus defined as IQR ≤ 1. Inferential analyses were conducted to assess differences in evaluator ratings across groups. One-way ANOVA was used to compare group means when the assumptions of normality and homogeneity of variance were reasonably met. A significance threshold of *P* ≤ .05 was applied, and effect sizes were calculated. To address potential deviations from these parametric assumptions and strengthen the validity of the findings, non-parametric tests were also performed, such as the Mann-Whitney U test for pairwise comparison. To provide additional context beyond *P*-values, we calculated effect sizes, including Eta-squared (η²) and Rank-biserial correlation for Mann-Whitney U tests. This approach ensured a methodologically rigorous and assumption-aware analysis of group differences. Statistical analyses were conducted using STATA® (STATA Corp., College Station, TX, USA).

## Results

In this study, 24 generated responses were analyzed across all 6 Bethesda categories, with each response assessed by 5 specialists for consistency, for a total of 120 evaluations (Table 1). The Likert mean score of consistency for all 24 responses was 3.38 out of 4, with a SD of 0.75. This corresponds to an overall rating of 75%-89% indicating moderate to good adherence to the guidelines. Response #5, “what does Bethesda II thyroid nodule mean”, demonstrated the highest mean score of 4.00 and is the only one rated as >90% consistent. On the other hand, responses #21 and #22 from Bethesda VI had the lowest mean scores of 2.60 out of 4, indicating 50%-74% consistency with the guidelines. Median consistency scores across individual responses ranged from 3.0 to 4.0, with an overall median of 3.5 (Table 1).

**Table 1.** Reponses Consistency with ATA Guidelines—Descriptive Statistics.

Question	Response	Count	Consensus	Mean	Median	SD	IQR
	#	(n)	Achieved				
I have a Bethesda I thyroid nodule.	R1-4	20	Yes	3.55	4	0.759	1
Q1: What does that mean?	R # 1	5	Yes	3.2	4	1.304	1
Q2: Do I have cancer?	R # 2	5	Yes	3.8	4	0.447	0
Q3: What are my options?	R # 3	5	Yes	3.8	4	0.447	0
Q4: What is the best option for me?	R # 4	5	Yes	3.4	3	0.548	1
I have a Bethesda II thyroid nodule.	R5-8	20	Yes	3.45	4	0.759	1
Q1: What does that mean?	R # 5	5	Yes	4	4	0.000	0
Q2: Do I have cancer?	R # 6	5	Yes	3.6	4	0.548	1
Q3: What are my options?	R # 7	5	Yes	3.4	3	0.548	1
Q4: What is the best option for me?	R # 8	5	Yes	2.8	3	1.095	0
I have a Bethesda III thyroid nodule.	R9-12	20	Yes	3.65	4	0.489	1
Q1: What does that mean?	R # 9	5	Yes	3.6	4	0.548	1
Q2: Do I have cancer?	R # 10	5	Yes	3.8	4	0.447	0
Q3: What are my options?	R # 11	5	Yes	3.6	4	0.548	1
Q4: What is the best option for me?	R # 12	5	Yes	3.6	4	0.548	1
I have a Bethesda IV thyroid nodule.	R13-16	20	Yes	3.35	3.5	0.745	1
Q1: What does that mean?	R # 13	5	Yes	3.4	3	0.548	1
Q2: Do I have cancer?	R # 14	5	Yes	3.2	3	0.837	1
Q3: What are my options?	R # 15	5	Yes	3.4	4	0.894	1
Q4: What is the best option for me?	R # 16	5	Yes	3.4	4	0.894	1
I have a Bethesda V thyroid nodule.	R17-20	20	Yes	3.3	3	0.470	1
Q1: What does that mean?	R # 17	5	Yes	3.4	3	0.548	1
Q2: Do I have cancer?	R # 18	5	Yes	3.4	3	0.548	1
Q3: What are my options?	R # 19	5	Yes	3.2	3	0.447	0
Q4: What is the best option for me?	R # 20	5	Yes	3.2	3	0.447	0
I have a Bethesda VI thyroid nodule.	R21-24	20	No	2.95	3	0.998	2
Q1: What does that mean?	R # 21	5	Yes	2.6	3	1.140	1
Q2: Do I have cancer?	R # 22	5	Yes	2.6	3	1.140	1
Q3: What are my options?	R # 23	5	No	3	3	1.000	2
Q4: What is the best option for me?	R # 24	5	Yes	3.6	4	0.548	1
Overall	R1-24	120	Yes	3.38	3.5	0.745	1

Data expressed as: N: number of evaluations performed; Mean and Median: ChatGPT’s responses assessed on a 4-point Likert scale: 1 point for consistency below 50% (low); 2 points for within the 50-74% range (low to moderate); 3 points for 75-89% (moderate to good); and 4 points for exceeding 90% (high); standard deviation (SD) and interquartile range (IQR); Consensus (RAND/UCLA Method) defined as IQR ≤ 1.

The distribution of expert ratings showed a clear concentration at the higher end of the scale, with most scores being 3 or 4 (109 out of 120), indicating that responses were commonly judged as consistent or highly consistent with clinical guidelines (Table 2). Consensus, defined by the RAND/UCLA Appropriateness Method as IQR ≤ 1, was achieved in 95.8% of responses, demonstrating strong inter-rater reliability (Table 2).

**Table 2.** Response Consistency—Code Distribution and Consensus Status.

Question	Response #	Count	1pt	2pts	3pts	4pts	Consensus
		(n)	< 50	50-74	75-89	≥ 90	Achieved
I have a Bethesda I thyroid nodule.	R1-4	20	1	0	6	13	Yes
Q1: What does that mean?	R # 1	5	1	0	1	3	Yes
Q2: Do I have cancer?	R # 2	5	0	0	1	4	Yes
Q3: What are my options?	R # 3	5	0	0	1	4	Yes
Q4: What is the best option for me?	R # 4	5	0	0	3	2	Yes
I have a Bethesda II thyroid nodule.	R5-8	20	1	0	8	11	Yes
Q1: What does that mean?	R # 5	5	0	0	0	5	Yes
Q2: Do I have cancer?	R # 6	5	0	0	2	3	Yes
Q3: What are my options?	R # 7	5	0	0	3	2	Yes
Q4: What is the best option for me?	R # 8	5	1	0	3	1	Yes
I have a Bethesda III thyroid nodule.	R9-12	20	0	0	7	13	Yes
Q1: What does that mean?	R # 9	5	0	0	2	3	Yes
Q2: Do I have cancer?	R # 10	5	0	0	1	4	Yes
Q3: What are my options?	R # 11	5	0	0	2	3	Yes
Q4: What is the best option for me?	R # 12	5	0	0	2	3	Yes
I have a Bethesda IV thyroid nodule.	R13-16	20	0	3	7	10	Yes
Q1: What does that mean?	R # 13	5	0	0	3	2	Yes
Q2: Do I have cancer?	R # 14	5	0	1	2	2	Yes
Q3: What are my options?	R # 15	5	0	1	1	3	Yes
Q4: What is the best option for me?	R # 16	5	0	1	1	3	Yes
I have a Bethesda V thyroid nodule.	R17-20	20	0	0	14	6	Yes
Q1: What does that mean?	R # 17	5	0	0	3	2	Yes
Q2: Do I have cancer?	R # 18	5	0	0	3	2	Yes
Q3: What are my options?	R # 19	5	0	0	4	1	Yes
Q4: What is the best option for me?	R # 20	5	0	0	4	1	Yes
I have a Bethesda VI thyroid nodule.	R21-24	20	2	4	7	7	No
Q1: What does that mean?	R # 21	5	1	1	2	1	Yes
Q2: Do I have cancer?	R # 22	5	1	1	2	1	Yes
Q3: What are my options?	R # 23	5	0	2	1	2	No
Q4: What is the best option for me?	R # 24	5	0	0	2	3	Yes
Overall	R1-24	120	4	7	49	60	Yes

Data expressed as: N: number of evaluations performed; ChatGPT’s responses assessed on a 4-point Likert scale: 1 point for consistency below 50% (low); 2 points for within the 50%-74% range (low to moderate); 3 points for 75%-89% (moderate to good); and 4 points for exceeding 90% (high); Consensus (RAND/UCLA Method) defined as IQR ≤ 1.

Analysis by Bethesda category (Table 3) revealed that 5 out of 6 categories were rated 75%-89% consistency, with mean Likert scores ranging from 3.30 to 3.65. Bethesda III had the highest mean score (3.65 out of 4) with a low SD (0.49). Conversely, Bethesda VI exhibited the lowest mean score (2.95 out of 4) and the highest SD (1.00), with 2 responses dipping the category’s performance into the 50%-74% range. Median consistency scores were highest (4.0) in Bethesda Categories I, II and III, and gradually declined in higher-risk categories —3.5 in Category IV, and 3.0 in Categories V and VI (Table 3). The overall median across all categories was 3.5, reflecting generally strong agreement, with slightly lower consensus in high-risk scenarios. The distribution of consistency scores showed that Codes 3 and 4 were the most frequently assigned across all categories. Consensus (IQR ≤ 1) was achieved in all but one category, with Bethesda VI showing a higher degree of variability among expert ratings and failing to meet the defined consensus threshold (Table 3). One-way ANOVA revealed a borderline significant difference in evaluator ratings across Bethesda categories (*P* = .05), with a small to moderate effect size (η² = 0.09), suggesting a potential trend in consistency ratings. A Mann-Whitney U test was conducted comparing low-risk (Bethesda I–III) and high-risk (Bethesda V–VI) categories. The results showed a statistically significant difference (*P* = .002), with a moderate effect size (rank-biserial correlation 0.323), indicating that responses associated with high-risk nodules were rated as significantly less consistent with clinical guidelines compared to those associated with low-risk nodules.

**Table 3.** ChatGPT’ Consistency across 6 Bethesda Categories.

Bethesda Category	Count	1pt	2pts	3pts	4pts	Consensus	Mean	Median	SD	IQR
	(n)	< 50	50-74	75-89	≥ 90	Achieved				
Overall	120	4	7	49	60	Yes	3.38	3.5	0.745	1
Bethesda I (R1-4)	20	1	0	6	13	Yes	3.55	4.0	0.759	1
Bethesda II (R2-8)	20	1	0	8	11	Yes	3.45	4.0	0.759	1
Bethesda III (R9-12)	20	0	0	7	13	Yes	3.65	4.0	0.489	1
Bethesda IV (R13-16)	20	0	3	7	10	Yes	3.35	3.5	0.745	1
Bethesda V (R17-20)	20	0	0	14	6	Yes	3.30	3.0	0.470	1
Bethesda VI (R20-24)	20	2	4	7	7	No	2.95	3.0	0.999	2

Data expressed as: N: number of evaluations performed; ChatGPT’s responses assessed on a 4-point Likert scale: 1 point for consistency below 50% (low); 2 points for within the 50%-74% range (low to moderate); 3 points for 75%-89% (moderate to good); and 4 points for exceeding 90% (high); Consensus (RAND/UCLA Method) defined as IQR ≤ 1.

When examining responses by question type across all Bethesda categories (Table 4), all four types achieved overall consistency ratings between 75% and 89%. Notably, responses corresponding to Question 3, “What are my options?,” were the most adherent with the guidelines, with a mean score of 3.40 and the lowest SD (0.68), while those for Questions 2, “Do I have cancer,” tie with Question 3 for the highest mean score but with a higher SD (0.77). Conversely, Responses to Question 4, “What is the best option for me?,” had the lowest mean score of 3.33; therefore, they were the least consistent with current guidelines. Median consistency scores across questions ranged from 3.0 for Question 4 to 4.0 for Questions 1 and 2, with an overall median score of 3.5. Consensus (IQR ≤ 1) was achieved for all questions, indicating uniform inter-rater agreement regardless of the question asked (Table 4). Inferential analyses revealed no statistically significant differences in evaluator ratings of consistency across the different questions. One-way ANOVA yielded a p-value of 0.984, indicating that mean consistency scores were highly similar regardless of the question. Pairwise Mann-Whitney U tests showed no significant differences between any question pairs (all *P*-values >.20), reinforcing the finding that the perceived conformity of AI-generated responses was consistent across all question types.

**Table 4.** Consistency Analysis by 4 Types of Questions Across Bethesda Categories.

Question Type	Count	1pt	2pts	3pts	4pts	Consensus	Mean	SD	Median	IQR
(Bethesda I-VI)	(n)	< 50	50-74	75-89	≥ 90	Achieved				
Overall	120	4	7	49	60	Yes	3.38	0.745	3.5	1
Q1	30	2	1	11	16	Yes	3.37	0.850	4.0	1
Q2	30	1	2	11	16	Yes	3.4	0.770	4.0	1
Q3	30	0	3	12	15	Yes	3.4	0.675	3.5	1
Q4	30	1	1	15	13	Yes	3.33	0.711	3.0	1

Question Type: The doctor said I have a Bethesda thyroid nodule. Q1: What does that mean? Q2: Do I have cancer? Q3 What are my options? Q4: What is the best option for me? ChatGPT’s responses assessed on a 4-point Likert scale: 1 point for consistency below 50% (low); 2 points for within the 50-74% range (low to moderate); 3 points for 75-89% (moderate to good); and 4 points for exceeding 90% (high); Consensus (RAND/UCLA Method) defined as IQR ≤ 1.

Specialists’ reluctance recommending ChatGPT to patients was lowest for Bethesda I thyroid nodules (mean score 1.45, SD 0.51), and highest for Bethesda V thyroid nodules (mean score 1.75, SD 0.51) (Table 5). The overall mean Likert score across the 6 Bethesda categories was 1.61 out of 2, with a standard deviation of 0.49. Median scores were consistently 2.0, with an IQR of 1.0 for all categories. All categories showed a mix of Code 1 (comfortable) and Code 2 (reluctant) ratings, with Code 2 being more frequent in high-risk categories. Despite this, consensus (IQR ≤ 1) was achieved across all categories, reflecting strong inter-rater agreement. Although there was no statistical difference between the groups (ANOVA *P*-value = .453; all pairwise comparisons *P* > .05), we could observe that clinicians’ reluctance to recommend the use of ChatGPT increased as the risk of malignancy in tumor nodules heightened.

**Table 5.** Clinicians’ Comfortability or Reluctance to Advise the Use of ChatGPT.

Bethesda Category	N	1pt Yes	2pts No	Consensus	Mean	Median	SD	IQR
Overall	120	47	73	Yes	1.61	2	0.490	1
Bethesda I (R 1-4)	20	11	9	Yes	1.45	1	0.5104	1
Bethesda II (R 2-8)	20	9	11	Yes	1.55	2	0.5104	1
Bethesda III (R 9-12)	20	8	12	Yes	1.6	2	0.5026	1
Bethesda IV (R 13-16)	20	8	12	Yes	1.6	2	0.5026	1
Bethesda V (R 17-20)	20	5	15	Yes	1.75	2	0.4443	0.25
Bethesda VI (R 20-24)	20	6	14	Yes	1.7	2	0.4702	1

Data expressed as: n: number of evaluations performed; Clinician’s comfortability or reluctance advising the use of ChatGPT as a clinical discussion complement assessed as follows: 1 point meaning “Yes, I would feel comfortable” 2 points meaning “No”; Consensus (RAND/UCLA Method) defined as IQR ≤ 1.

**Figure 1.**
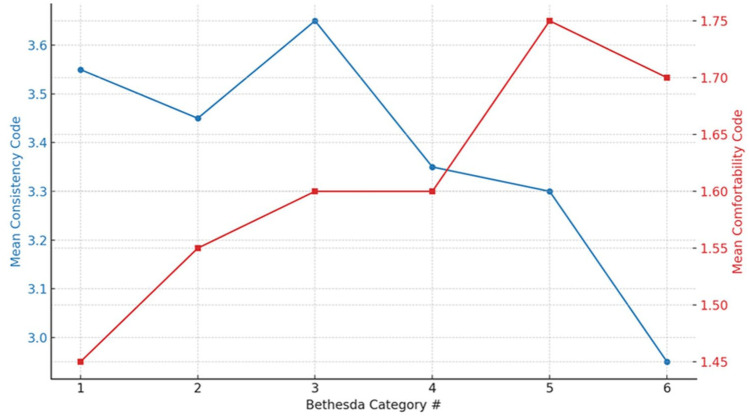
Trend in mean scores across all 6 Bethesda categories. Line blue represents Consistency scores and line red represents Comfortability scores.

Figure 1 compares mean trends across all 6 Bethesda risk categories, revealing a decrease in response consistency and a corresponding increase in clinician reluctance as risk for malignancy increase.

## Discussion

Thyroid nodule diagnostic tools, including fine needle aspiration (FNA), are associated with increased psychological distress and sleep disturbances in patients.^8^ As a result, concerned patients often turn to the Internet to supplement their discussions with clinicians. Nevertheless, studies have also uncovered that many individuals experience frustration and difficulty when accessing health information online. In addition, many online sources about thyroid conditions are written at grade levels above the recommended reading level.^9,10^ In contrast, ChatGPT rapidly compiles information from available literature and presents it in a conversational manner, using simplified vocabulary.^7^

Our results indicate that ChatGPT’s recommendations for thyroid nodule management are moderately consistent with current guidelines for Bethesda IV, V, and VI nodules and highly consistent for Bethesda I, II, and III nodules. We also found a significant association between the consistency of ChatGPT's responses with the ATA guidelines and other similar guidelines and clinicians' reluctance to recommend its use. Notably, clinicians were more comfortable recommending ChatGPT for patients with lower-risk Bethesda categories (I–III) and less so for higher-risk categories (V-VI). This inverse relationship suggests that as the complexity and potential severity of the clinical scenario increase, so does the caution exercised by healthcare professionals in relying on AI-generated advice.

Despite ChatGPT’s lower consistency with management guidelines for Bethesda IV, V, and VI nodules, our evaluation of its responses suggests that it is generally a safe tool for patients to use because it consistently recommends seeking further professional care. Our findings across Bethesda categories align with those of a study by Koroglu et al., in which a large language model–based chatbot (such as ChatGPT) demonstrated correctness and reliability scores ranging from 6.09 to 6.47 out of 7.^11^ A study by Campbell et al. further supports our results, reporting that despite differences in language level when prompting ChatGPT on thyroid nodule management, the chatbot provided mostly correct answers, though some hallucinations were noted.^12^

Furthermore, ChatGPT demonstrates a high diagnostic ability, providing answers that are highly consistent with current guidelines when addressing questions such as “Do I have cancer?” and “What are my options?”. However, the chatbot is less consistent when answering questions that require clinical interpretation, such as “What does that mean?” and “What is the best option for me?.” A technical report by OpenAI on ChatGPT-4 and ChatGPT-3.5 highlights the chatbot’s limitations in logical reasoning and its tendency to produce “hallucinations,” or incorrect information, due to a verification failure.^13^ This inability to extrapolate accurately may contribute to ChatGPT’s lower consistency with higher Bethesda scores.

Our study highlights both the potential and limitations of ChatGPT in providing accurate recommendations for thyroid nodule management based on TBSRTC. While the chatbot demonstrated a moderate to good consistency with current guidelines, several key issues emerged that warrant further discussion.

Firstly, it is crucial to recognize that ChatGPT's responses are subject to variability based on the phrasing of questions and follow-up statements. Different wordings and follow-up questions can lead to different outcomes, often resulting in refined responses ^13^. This variability underscores the need for precise and carefully crafted prompts to achieve the most accurate and helpful answers. Proper prompt engineering and detailed prompts are often associated with better accuracy, suggesting that users need to be trained in effective querying techniques to maximize the utility of AI models, such as ChatGPT.^13^

ChatGPT, as a conversational AI model powered by natural language processing technology, can struggle to fully capture the nuances of complex medical queries ^13,14^. This limitation is particularly evident in its tendency to provide lengthy responses that may hinder comprehension. For example, when tasked with explaining the implications of a Bethesda category, ChatGPT often fails to deliver concise, easily digestible information. This raises concerns about its effectiveness in clinical settings, where clarity and brevity are paramount.^15^

The study also raises the question of whether ChatGPT’s performance would differ in more complex, scenario-based assessments, such as clinical vignettes or Objective Structured Clinical Examinations (OSCEs), compared to straightforward, guidelines-based questions.^16^ Further research is needed to explore ChatGPT's capabilities in these more nuanced contexts. Literature suggests that accuracy tends to be higher when responses are based on guidelines, followed by patient education materials, OSCE questions, and clinical vignettes, in descending order of accuracy.^16,17^ This pattern indicates that the complexity of the clinical scenario might influence ChatGPT's performance.

Importantly, this study underscores clinicians’ reluctance to recommend ChatGPT-generated responses for higher-risk Bethesda categories, where diagnostic ambiguity and management decisions require deeper clinical reasoning. This hesitancy likely reflects ongoing concerns that, in complex or high-stakes scenarios, AI tools may introduce oversimplifications, particularly in contexts that demand nuanced interpretation and expert clinical insight. Existing literature suggests that, while AI holds promise as a decision-support tool, it currently lacks the contextual awareness and clinical intuition needed to manage cases involving malignancy risk stratification or individualized treatment planning.^18^ Given these current limitations, clinicians may remain cautious about viewing AI as a reliable complement in complex, high-risk clinical decision-making—though this perspective may shift as the technology matures.

Another important consideration is the type of knowledge required to answer specific questions. Declarative knowledge, which includes concrete, verifiable facts such as dates, definitions, and formulas, is where ChatGPT excels.^16,17^ This type of knowledge is binary in nature, with definitive correctness or incorrectness. In contrast, contextual knowledge requires navigating multiple variables and shades of gray, involving higher-order cognitive skills like analysis and evaluation. This study showed that while ChatGPT performed well with declarative knowledge as reflected in guideline-based questions, its performance in more contextually complex scenarios like clinical vignettes may be less reliable.

Overall, while ChatGPT shows promise as a supportive tool in the management of thyroid nodules, particularly in cases involving lower Bethesda scores, several challenges remain. The AI’s ability to handle complex clinical scenarios, provide concise information, and stay updated with the latest guidelines needs further refinement. Future advancements in AI technology may enhance its accuracy and reliability, making it a more viable option for clinical decision-making. However, for now, ChatGPT should be used cautiously and as a complement to, rather than a replacement for, professional medical consultation.

## Limitations

The principal limitation of our study is ChatGPT’s inherent machine learning capabilities. This allows its knowledge base to continually expand as more scientific literature emerges. However, this does not guarantee that its responses will always reflect the latest medical guidelines on thyroid nodule management, depending on what Internet source is available or prioritized in its programming.^13^ In addition, Chatbot may not always capture the context of a prompt and the nuances of a query.^13^ Integrating follow-up prompts could refine ChatGPT’s responses, leading to more targeted information and effectively reducing bias.

Another limitation of this study arises from the language in which the queries were conducted. As all interactions with ChatGPT were carried out in English, the results may not be universally applicable to patients with thyroid nodules who seek care in other languages.^19,20^ This linguistic constraint could influence the reproducibility and applicability of our findings across diverse linguistic demographics. Further research is necessary to evaluate conversational AI’s performance in responding to queries in other languages, which would help in ensuring its broader application and utility in a global healthcare context.^20^

Several methodological limitations should be acknowledged. First, panel composition: Although the panel consisted of highly specialized thyroid surgeons and endocrinologists, the absence of broader multidisciplinary representation may limit the generalizability of the findings. Future studies should aim to include a more diverse group of evaluators to reduce specialty-related bias and strengthen the validity of consensus-based assessments. Exclusion of additional clinical parameters: Important variables such as nodule size, ultrasound characteristics, and molecular testing—known to influence risk stratification and clinical decision-making—were intentionally omitted to focus on ChatGPT’s ability to respond based solely on Bethesda classification. While this approach facilitated a standardized assessment, it inherently limited the clinical complexity and risk variability represented in the scenarios. Future research should incorporate these parameters to assess the AI’s ability to adapt responses to complex, parameter-rich clinical contexts reflective of real-world decision-making.

## Conclusion

ChatGPT’s answers, in order of most to least consistent with the current guidelines, including from the ATA, are those for Bethesda categories III, I, II, IV, V, and VI. Inversely, experienced thyroid specialists feel most to least comfortable recommending the use of ChatGPT-3.5 as a clinical discussion complement to patients affected by Bethesda I, Bethesda II, Bethesda III and IV, Bethesda VI and Bethesda V thyroid nodules. Also, ChatGPT-3.5 provides its most reliable answers for descriptive questions, whereas its reliability decreases when answering clinical interpretation questions. Our findings underscore the potential of ChatGPT as a supportive tool in thyroid nodule management, particularly in scenarios with lower Bethesda scores. However, challenges persist, especially in addressing complex cases involving thyroid nodules with higher Bethesda scores, suggesting the need for further refinement and validation of AI-driven systems, such as ChatGPT, in clinical decision-making processes. Nevertheless, with its promising abilities, further advancements in AI technology may pave the way for improved accuracy and reliability in the future.

## Supplemental Material

sj-docx-1-ohn-10.1177_19160216251387617 – Supplemental material for Thyroid Nodule Experts Evaluating ChatGPT’s Assessment of Thyroid Nodules Classified by the Bethesda System for Reporting Thyroid Cytopathology Supplemental material, sj-docx-1-ohn-10.1177_19160216251387617 for Thyroid Nodule Experts Evaluating ChatGPT’s Assessment of Thyroid Nodules Classified by the Bethesda System for Reporting Thyroid Cytopathology by Alexander Moise, Luiza Tatar, Noa Sela, Sabrina Daniela da Silva, Jasmine Kouz, Michael Tamilia, Michael P. Hier, Veronique-Isabelle Forest and Richard J. Payne in Journal of Otolaryngology - Head & Neck Surgery

## References

[1] Ali SZ Baloch ZW Cochand-Priollet B Schmitt FC Vielh P VanderLaan PA. The 2023 Bethesda system for reporting thyroid cytopathology. Thyroid. 2023;33(9):1039-1044. doi:10.1089/thy.2023.0141 37427847

[2] Haugen BR Alexander EK Bible KC , et al. 2015 American thyroid association management guidelines for adult patients with thyroid nodules and differentiated thyroid cancer: The American Thyroid Association guidelines task force on thyroid nodules and differentiated thyroid Cancer. Thyroid. Jan 2016;26(1):1-133. doi:10.1089/thy.2015.0020 26462967 PMC4739132

[3] Johnson D Goodman R Patrinely J , et al. Assessing the accuracy and reliability of AI-generated medical responses: an evaluation of the Chat-GPT model. Res Sq. 2023;doi:10.21203/rs.3.rs-2566942/v1

[4] Kung TH Cheatham M Medenilla A , et al. Performance of ChatGPT on USMLE: Potential for AI-assisted medical education using large language models. PLOS Digit Health. 2023;2(2):e0000198. doi:10.1371/journal.pdig.0000198 PMC9931230 36812645

[5] Lechien JR Chiesa-Estomba CM Baudouin R Hans S. Accuracy of ChatGPT in head and neck oncological board decisions: preliminary findings. Eur Arch Otorhinolaryngol. 2023;281:2105-2114. doi:10.1007/s00405-023-08326-w 37991498

[6] Mondal H Dash I Mondal S Behera JK. ChatGPT in Answering Queries Related to Lifestyle-Related Diseases and Disorders. Cureus. 2023;15(11):e48296. doi:10.7759/cureus.48296 PMC10696911 38058315

[7] Dave T Athaluri SA Singh S. ChatGPT in medicine: an overview of its applications, advantages, limitations, future prospects, and ethical considerations. Front Artif Intell. 2023;6:1169595. doi:10.3389/frai.2023.1169595 37215063 PMC10192861

[8] Li R Li G Wang Y , et al. Psychological distress and sleep disturbance throughout thyroid nodule screening, diagnosis, and treatment. J Clin Endocrinol Metab. Sep 27 2021;106(10):e4221-e4230. doi:10.1210/clinem/dgab224 33830242

[9] Finney Rutten LJ Blake KD , et al. Online health information seeking among US adults: measuring progress toward a healthy people 2020 objective. Public Health Rep. 2019;134(6):617-625. doi:10.1177/0033354919874074 31513756 PMC6832079

[10] Cimbek EA Cimbek A. Online health information on thyroid nodules: do patients understand them? Minerva Endocrinol (Torino). 2023;50:144-150. doi:10.23736/S2724-6507.23.03952-0 37021923

[11] Koroglu EY Faki S Bestepe N , et al. A novel approach: evaluating ChatGPT’s utility for the management of thyroid nodules. Cureus. 2023;15(10):e47576. doi:10.7759/cureus.47576 PMC10666652 38021609

[12] Campbell DJ Estephan LE Sina EM , et al. Evaluating ChatGPT responses on thyroid nodules for patient education. Thyroid. 2024;34(3):371-377. doi:10.1089/thy.2023.0491 38010917

[13] OpenAI. ChatGPT-4 Technical Report. Accessed June 1, 2024. https://cdn.openai.com/papers/gpt-4.pdf

[14] Yeo YH Samaan JS Ng WH , et al. Assessing the performance of ChatGPT in answering questions regarding cirrhosis and hepatocellular carcinoma. Clin Mol Hepatol. 2023;29(3):721-732. doi:10.3350/cmh.2023.0089 36946005 PMC10366809

[15] Ghosh AK Joshi S Ghosh A. Effective patient-physician communication - a concise review. J Assoc Physicians India. 2020;68(6):53-57. 32610880

[16] Massey PA Montgomery C Zhang AS. Comparison of ChatGPT-3.5, ChatGPT-4, and orthopaedic resident performance on orthopaedic assessment examinations. J Am Acad Orthop Surg. 2023;31(23):1173-1179. doi:10.5435/JAAOS-D-23-00396 37671415 PMC10627532

[17] Antaki F Touma S Milad D El-Khoury J Duval R. Evaluating the performance of ChatGPT in Ophthalmology: an analysis of its successes and shortcomings. Ophthalmol Sci. 2023;3(4):100324. doi:10.1016/j.xops.2023.100324 37334036 PMC10272508

[18] Matuchansky C. Deep medicine, artificial intelligence, and the practising clinician. The Lancet. 2019;394(10200):736. doi:10.1016/S0140-6736(19)31235-8 31478500

[19] Soto-Chavez MJ Bustos MM Fernandez-Avila DG Munoz OM. Evaluation of information provided to patients by ChatGPT about chronic diseases in Spanish language. Digit Health. 2024;10:20552076231224603. doi:10.1177/20552076231224603 PMC10768597 38188865

[20] Yeo YH Samaan JS Ng WH , et al. GPT-4 outperforms ChatGPT in answering non-English questions related to cirrhosis. medRxiv. 2023.05.04.23289482. doi: 10.1101/2023.05.04.23289482

