# Supplementary material for: Thyroid Nodule Experts Evaluating ChatGPT’s Assessment of Thyroid Nodules Classified by the Bethesda System for Reporting Thyroid Cytopathology
Source: J Otolaryngol Head Neck Surg. 2025 Dec 1;54:19160216251387617. doi: 10.1177/19160216251387617 (PMC12669496; doi:10.1177/19160216251387617)
Supplement: sj-docx-1-ohn-10.1177_19160216251387617 – Supplemental material for Thyroid Nodule Experts Evaluating ChatGPT’s Assessment of Thyroid Nodules Classified by the Bethesda System for Reporting Thyroid Cytopathology [file sj-docx-1-ohn-10.1177_19160216251387617.docx]

**Table 1. ChatGPT Responses to Questions Regarding the Management of Thyroid Nodules**

| **Question** | **Response** |
| --- | --- |
| I have a Bethesda I thyroid nodule. | R1-4 |
| Q1: What does that mean? | It seems like there might be a bit of confusion or a misunderstanding. The Bethesda System for Reporting Thyroid Cytopathology (TBSRTC) is a standardized system used to report the results of thyroid fine needle aspiration (FNA) biopsies, which are commonly done to evaluate thyroid nodules. The Bethesda System categorizes the findings into six main diagnostic categories, ranging from benign to malignant. These categories help guide further management and determine the need for additional testing or surgery.  The categories are: 1. **Non-diagnostic or Unsatisfactory:** The sample does not provide enough information for a diagnosis. 2. **Benign:** The cells in the sample are consistent with a benign (non-cancerous) nodule. 3. **Atypia of Undetermined Significance (AUS) or Follicular Lesion of Undetermined Significance (FLUS):** The cells show some abnormalities, but the significance is uncertain. 4. **Follicular Neoplasm or Suspicious for a Follicular Neoplasm:** The cells suggest a possible follicular neoplasm, which may be benign or malignant. 5. **Suspicious for Malignancy:** The cells indicate a higher likelihood of malignancy. 6. **Malignant:** The cells are consistent with a malignant (cancerous) nodule.  If your doctor mentioned a "Bethesda I thyroid nodule," it might be a miscommunication or a misinterpretation. Bethesda I typically refers to the category of "Non-diagnostic or Unsatisfactory," where the sample obtained during the FNA does not provide enough information for a definitive diagnosis.  It's essential to discuss the results further with your doctor to clarify the findings, understand the implications, and determine the next steps in your medical care. They can provide more information about the specific characteristics of the nodule and guide you through any additional tests or treatments that may be necessary. |
| Q2: Do I have cancer? | A Bethesda I thyroid nodule means that the sample obtained during the fine needle aspiration (FNA) biopsy was non-diagnostic or unsatisfactory, and it does not provide enough information for a definitive diagnosis. The Bethesda System for Reporting Thyroid Cytopathology (TBSRTC) categorizes FNA results into six main diagnostic categories, ranging from non-diagnostic to malignant.  In the Bethesda System:  Bethesda I: Non-diagnostic or Unsatisfactory  This category indicates that the sample collected during the biopsy did not yield enough cellular material or the material obtained was not suitable for interpretation. It does not provide information about whether the nodule is benign or malignant.  Having a Bethesda I result does not necessarily mean you have cancer. In many cases, a repeat FNA or alternative diagnostic procedures may be recommended to obtain a more conclusive sample for analysis. Your healthcare provider will guide you on the appropriate next steps based on your individual case.  It's crucial to follow up with your doctor to discuss the results, understand the implications, and determine the best course of action for further evaluation. Additional tests or imaging studies may be recommended to gather more information about the thyroid nodule and to determine whether it is benign or potentially cancerous. |
| Q3: What are my options? | If you have received a Bethesda I thyroid nodule result, which indicates that the sample obtained during the fine needle aspiration (FNA) biopsy was non-diagnostic or unsatisfactory, there are several potential options and considerations:  1.**Repeat FNA:** One common approach is to repeat the FNA procedure. This may involve obtaining a new sample from the thyroid nodule to improve the chances of obtaining sufficient cellular material for a diagnosis. The repeat FNA may be done using ultrasound guidance to target the nodule more accurately.  2.**Diagnostic Imaging:** Your healthcare provider may recommend additional imaging studies, such as ultrasound, to further evaluate the thyroid nodule. Imaging can provide information about the size, characteristics, and features of the nodule, helping to guide the next steps in your care.  3.**Clinical Observation:** Depending on the clinical scenario and the characteristics of the thyroid nodule, your doctor may opt for a period of observation. This could involve monitoring the nodule over time through regular follow-up visits and imaging studies to assess any changes in size or appearance.  4.**Consideration of Alternative Testing:** In some cases, alternative diagnostic tests, such as molecular testing or genetic analysis of the thyroid nodule, may be considered to gather more information about the nature of the nodule.  5.**Consultation with a Specialist:** Depending on the complexity of your case, your healthcare provider may refer you to an endocrinologist or a thyroid specialist for further evaluation and management.  It's important to have a detailed discussion with your healthcare provider to understand your specific situation, the characteristics of the thyroid nodule, and the most appropriate course of action. They can provide guidance on the available options, discuss the potential risks and benefits of each approach, and help you make informed decisions about your care. Regular follow-up and communication with your healthcare team are key aspects of managing thyroid nodules and ensuring appropriate care. |
| Q4: What is the best option for me? | The best option for managing a Bethesda I thyroid nodule depends on various factors, including your individual medical history, the characteristics of the nodule, and your overall health. Here are some considerations and potential options:  1.**Repeat Fine Needle Aspiration (FNA):** Since Bethesda I indicates that the initial FNA result was non-diagnostic or unsatisfactory, one option is to undergo a repeat FNA. This may provide a new sample for analysis, with the goal of obtaining sufficient cellular material for a more conclusive diagnosis.  2.**Diagnostic Imaging:** Your healthcare provider may recommend additional imaging studies, such as ultrasound, to further evaluate the thyroid nodule. Imaging can help assess the size, characteristics, and features of the nodule, aiding in decision-making about the next steps.  3.**Clinical Observation:** Depending on the clinical context and the characteristics of the nodule, your doctor might choose a period of observation. This involves monitoring the nodule over time through regular follow-up visits and imaging studies to assess any changes.  4.**Consultation with a Specialist:** Depending on the complexity of your case, your healthcare provider may refer you to an endocrinologist or a thyroid specialist for further evaluation and management. Specialists can provide expertise in thyroid disorders and help guide decisions about additional testing or treatment.  5.**Consideration of Alternative Testing:** In some cases, your healthcare provider might recommend alternative diagnostic tests, such as molecular testing or genetic analysis of the thyroid nodule, to gather more information about its nature.  It's essential to have a thorough discussion with your healthcare provider to understand your specific situation, the characteristics of the thyroid nodule, and the available options. They can help you weigh the risks and benefits of each approach, taking into account your overall health and preferences.  If you haven't already, consider asking your healthcare provider the following questions:  - What are the characteristics of the thyroid nodule?  - What are the potential risks and benefits of repeat FNA, imaging studies, or other diagnostic tests?  - What is the recommended course of action based on my individual case?  Remember that open communication with your healthcare team is crucial for making informed decisions about your care. |
| I have a Bethesda II thyroid nodule. | R5-8 |
| Q1: What does that mean? | A Bethesda II thyroid nodule refers to a specific category in the Bethesda System for Reporting Thyroid Cytopathology (TBSRTC). This system is used to classify the results of thyroid fine needle aspiration (FNA) biopsies, which are commonly performed to evaluate thyroid nodules. The Bethesda System categorizes the findings into six main diagnostic categories to guide further management. Bethesda II is one of these categories.  Here's what Bethesda II means:  **Bethesda II - Benign:**  This category indicates that the cells collected from the thyroid nodule during the FNA biopsy are considered benign, meaning non-cancerous. It suggests that the nodule is likely not cancerous, and no further immediate action may be needed for malignancy concerns.  However, it's important to note that even though Bethesda II is associated with a benign interpretation, the final diagnosis should be considered in the broader clinical context, and your healthcare provider will guide you on the appropriate next steps.  Your doctor may recommend continued monitoring of the nodule, especially if it's large or causing symptoms. Follow-up imaging or periodic evaluations may be suggested to ensure the stability of the nodule over time.  It's crucial to discuss the results and any recommended follow-up with your healthcare provider to understand your specific situation and receive guidance on managing the thyroid nodule. |
| Q2: Do I have cancer? | A Bethesda II thyroid nodule indicates that the cells obtained from the fine needle aspiration (FNA) biopsy are benign, meaning they are not cancerous. In the Bethesda System for Reporting Thyroid Cytopathology, each category provides information about the likelihood of malignancy, and Bethesda II is associated with a benign interpretation.  Having a Bethesda II result means that, based on the examination of the sample, there is no evidence of cancer in the thyroid nodule. However, it's essential to understand that while Bethesda II is reassuring for the absence of malignancy, it doesn't guarantee that the nodule won't change over time or that other thyroid conditions may not be present.  Your healthcare provider will guide you on the appropriate next steps based on your individual case. In many instances, they may recommend continued monitoring of the nodule, especially if it's large or causing symptoms. Follow-up imaging or periodic evaluations may be suggested to ensure the stability of the nodule over time.  It's crucial to maintain open communication with your healthcare team, attend follow-up appointments as recommended, and report any new symptoms or changes in the nodule to ensure ongoing monitoring of your thyroid health. |
| Q3: What are my options? | If you have received a Bethesda II thyroid nodule result, indicating a benign interpretation based on the fine needle aspiration (FNA) biopsy, it means that there is no evidence of cancer in the nodule. Your healthcare provider will guide you on the appropriate next steps based on your individual case, but here are some common considerations and options:  1. **Observation and Follow-Up:**  - If the nodule is small and not causing significant symptoms, your doctor may recommend a watchful waiting approach. This involves regular monitoring of the nodule through follow-up appointments and possibly repeat imaging studies to ensure stability.  2. **Additional Imaging Studies:**  - Your healthcare provider may recommend additional imaging studies, such as ultrasound, to further assess the characteristics of the nodule and monitor for any changes over time.  3. **Thyroid Hormone Therapy:**  - In some cases, especially if the nodule is associated with thyroid hormone imbalances, your doctor may prescribe thyroid hormone replacement therapy to address thyroid function and potentially reduce the size of the nodule.  4. **Consultation with a Specialist:**  - Depending on the complexity of your case, your healthcare provider may refer you to an endocrinologist or a thyroid specialist for further evaluation and management.  5. **Patient Education and Lifestyle Management:**  - Your healthcare provider may provide information on thyroid health, lifestyle factors, and dietary considerations to optimize your overall well-being.  It's important to have a detailed discussion with your healthcare provider to understand your specific situation and to address any questions or concerns you may have. Be sure to ask about the recommended follow-up schedule and reporting any new symptoms or changes in the nodule.  Remember that individual cases can vary, and the best course of action will depend on your unique circumstances. Regular communication with your healthcare team is essential for ongoing thyroid health management. |
| Q4: What is the best option for me? | ChatGPT's answer: A Bethesda II thyroid nodule result is generally reassuring, as it indicates that the cells obtained from the fine needle aspiration (FNA) biopsy are benign (non-cancerous). In such cases, the best option for you will depend on various factors, including the characteristics of the nodule, your overall health, and your preferences. Here are some common options:  1. **Observation and Follow-Up:**  - If the nodule is small, not causing significant symptoms, and has benign characteristics, your healthcare provider may recommend a watchful waiting approach. This involves periodic monitoring through follow-up appointments and imaging studies to ensure the stability of the nodule.  2. **Thyroid Hormone Therapy:**  - If the nodule is associated with thyroid hormone imbalances or other thyroid conditions, your doctor may prescribe thyroid hormone replacement therapy. This can help regulate thyroid function and may, in some cases, lead to a reduction in the size of the nodule.  3. **Lifestyle Modifications:**  - Your healthcare provider may provide guidance on lifestyle factors that can contribute to overall thyroid health, such as a balanced diet, regular exercise, and stress management.  4. **Education and Counseling:**  - Understanding your thyroid health is crucial. Your healthcare provider may provide education about thyroid conditions, the significance of benign nodules, and any lifestyle modifications that can promote thyroid health.  5. **Consultation with a Specialist:**  - Depending on the specific characteristics of the nodule or if there are other thyroid-related concerns, your healthcare provider might refer you to an endocrinologist or a thyroid specialist for further evaluation and management.  It's essential to discuss your specific case with your healthcare provider to determine the most appropriate course of action. Ask questions about the characteristics of the nodule, the recommended follow-up schedule, and any signs or symptoms to watch for. If you have concerns or uncertainties, seeking a second opinion from another healthcare professional or specialist may also be an option.  Remember that ongoing communication with your healthcare team is key to ensuring that your thyroid health is effectively managed. |
| I have a Bethesda III thyroid nodule. | R9-12 |
| Q1: What does that mean? | A Bethesda III thyroid nodule refers to a specific category in the Bethesda System for Reporting Thyroid Cytopathology (TBSRTC). This system is used to classify the results of thyroid fine needle aspiration (FNA) biopsies, which are commonly performed to evaluate thyroid nodules. The Bethesda System categorizes the findings into six main diagnostic categories to guide further management. Bethesda III is one of these categories.  Here's what Bethesda III means:  **Bethesda III - Atypia of Undetermined Significance or Follicular Lesion of Undetermined Significance (AUS/FLUS):**  This category indicates that the cells obtained from the FNA biopsy show some atypical features, but the significance is uncertain. The findings are not definitive for either a benign or malignant diagnosis. The term "atypia" implies that the cells do not appear entirely normal but fall short of meeting criteria for a more definitive classification.  When a Bethesda III result is reported, the next steps in management will typically involve additional evaluation to better characterize the nature of the thyroid nodule and determine the risk of malignancy.  Your healthcare provider may recommend further tests, such as repeat FNA, molecular testing, or additional imaging studies, to gather more information about the nodule. A consultation with an endocrinologist or a thyroid specialist might also be considered for a more comprehensive evaluation.  It's crucial to have a detailed discussion with your healthcare provider to understand your specific situation, the characteristics of the thyroid nodule, and the recommended next steps. Additional testing and close monitoring are often necessary to reach a more definitive diagnosis and guide appropriate treatment decisions. |
| Q2: Do I have cancer? | A Bethesda III thyroid nodule result (Atypia of Undetermined Significance or Follicular Lesion of Undetermined Significance - AUS/FLUS) indicates that the cells obtained from the fine needle aspiration (FNA) biopsy show some atypical features, but the significance is uncertain. This category is indeterminate and does not provide a clear distinction between benign and malignant findings.  The Bethesda III category carries a variable risk of malignancy, and the likelihood of cancer depends on several factors, including the specific characteristics of the atypical cells, the size of the nodule, and other clinical factors. The risk of malignancy for Bethesda III nodules is typically in the range of 5% to 15%, but it can vary.  To further assess the risk and make a more definitive diagnosis, your healthcare provider may recommend additional tests or procedures, including:  1. **Repeat FNA:** A repeat fine needle aspiration may be performed to obtain a new sample for analysis, hoping to provide more conclusive results.  2. **Molecular Testing:** Molecular testing of the FNA sample may be considered to assess genetic or molecular markers associated with malignancy.  3. **Imaging Studies:** Additional imaging studies, such as ultrasound or other diagnostic scans, may be used to evaluate the characteristics of the nodule and surrounding tissues.  4. **Consultation with a Specialist:** Your healthcare provider may refer you to an endocrinologist or a thyroid specialist for a more in-depth evaluation and to discuss further management options.  It's important to note that a Bethesda III result does not necessarily mean that you have cancer, but further investigation is needed to determine the nature of the thyroid nodule. Discuss the findings and recommended next steps with your healthcare provider to develop an appropriate plan for further evaluation and management based on your individual case. |
| Q3: What are my options? | If you have received a Bethesda III thyroid nodule result, indicating Atypia of Undetermined Significance or Follicular Lesion of Undetermined Significance (AUS/FLUS), your healthcare provider will likely discuss further options and evaluations to better characterize the nature of the nodule. Here are common options:  1. **Repeat Fine Needle Aspiration (FNA):**  - A repeat FNA biopsy may be recommended to obtain a new sample for analysis. This is done to improve the chances of obtaining sufficient cellular material for a more conclusive diagnosis.  2. **Molecular Testing:**  - Molecular testing of the FNA sample may be considered to assess genetic or molecular markers associated with malignancy. This can provide additional information to help determine the risk of cancer.  3. **Imaging Studies:**  - Additional imaging studies, such as ultrasound or other diagnostic scans, may be used to evaluate the characteristics of the nodule and surrounding tissues. Imaging can provide valuable information about the size, shape, and features of the nodule.  4. **Consultation with a Specialist:**  - Your healthcare provider may refer you to an endocrinologist or a thyroid specialist for a more in-depth evaluation. Specialists can provide expertise in thyroid disorders and help guide decisions about additional testing or treatment.  5. **Clinical Observation:**  - In some cases, especially if the nodule is small and not causing significant symptoms, your healthcare provider may recommend a period of observation. This involves regular monitoring through follow-up appointments and imaging studies to assess any changes in the nodule over time.  It's important to have a detailed discussion with your healthcare provider to understand your specific situation and to address any questions or concerns you may have. Be sure to discuss the recommended next steps, the potential risks and benefits of each option, and the expected timeline for follow-up and further evaluation.  Thyroid nodules in the Bethesda III category present a challenge in terms of diagnosis, and additional testing is typically required to determine the appropriate course of action based on your individual case. Regular communication with your healthcare team is crucial for making informed decisions about your care. |
| Q4: What is the best option for me? | The best option for managing a Bethesda III thyroid nodule (Atypia of Undetermined Significance or Follicular Lesion of Undetermined Significance - AUS/FLUS) depends on several factors, including the specific characteristics of the nodule, your overall health, and your preferences. Here are potential options that your healthcare provider may consider:  1. **Repeat Fine Needle Aspiration (FNA):**  - A repeat FNA biopsy may be recommended to obtain a new sample for analysis. This is done to improve the chances of obtaining sufficient cellular material for a more conclusive diagnosis.  2. **Molecular Testing:**  - Molecular testing of the FNA sample may be considered to assess genetic or molecular markers associated with malignancy. This can provide additional information to help determine the risk of cancer.  3. **Imaging Studies:**  - Additional imaging studies, such as ultrasound or other diagnostic scans, may be used to evaluate the characteristics of the nodule and surrounding tissues. Imaging can provide valuable information about the size, shape, and features of the nodule.  4. **Clinical Observation:**  - In some cases, especially if the nodule is small and not causing significant symptoms, your healthcare provider may recommend a period of observation. This involves regular monitoring through follow-up appointments and imaging studies to assess any changes in the nodule over time.  5. **Consultation with a Specialist:**  - Your healthcare provider may refer you to an endocrinologist or a thyroid specialist for a more in-depth evaluation. Specialists can provide expertise in thyroid disorders and help guide decisions about additional testing or treatment.  The specific recommendation will be based on the unique characteristics of your case. It's important to have a detailed discussion with your healthcare provider to understand your specific situation, the potential risks and benefits of each option, and the expected timeline for follow-up and further evaluation.  Thyroid nodules in the Bethesda III category present a diagnostic challenge, and your healthcare team will work with you to determine the most appropriate course of action based on your individual circumstances. Regular communication with your healthcare team is crucial for making informed decisions about your care. |
| I have a Bethesda IV thyroid nodule. | R13-16 |
| Q1: What does that mean? | A Bethesda IV thyroid nodule refers to a specific category in the Bethesda System for Reporting Thyroid Cytopathology (TBSRTC). This system is used to classify the results of thyroid fine needle aspiration (FNA) biopsies, which are commonly performed to evaluate thyroid nodules. The Bethesda System categorizes the findings into six main diagnostic categories to guide further management, and Bethesda IV is one of these categories.  Here's what Bethesda IV means:  **Bethesda IV - Follicular Neoplasm or Suspicious for a Follicular Neoplasm:**  This category indicates that the cells obtained from the FNA biopsy show features suggestive of a follicular neoplasm, which includes both benign and potentially malignant conditions. The distinction between benign and malignant follicular neoplasms cannot be made definitively based on the FNA results alone.  When a Bethesda IV result is reported, further evaluation is typically needed to determine the nature of the follicular neoplasm and whether surgical intervention is necessary.  Your healthcare provider may recommend additional tests or procedures, such as:  1. **Molecular Testing:** Molecular testing of the FNA sample may be considered to assess genetic or molecular markers associated with malignancy. This can provide additional information to help determine the risk of cancer.  2. **Thyroid Imaging:** Additional imaging studies, such as ultrasound or other diagnostic scans, may be used to evaluate the characteristics of the nodule and surrounding tissues.  3. **Surgical Consultation:** Due to the indeterminate nature of Bethesda IV results, your healthcare provider may refer you to a thyroid surgeon for a surgical consultation. In some cases, surgical removal of the nodule or a portion of the thyroid may be recommended for a definitive diagnosis.  It's important to have a detailed discussion with your healthcare provider to understand your specific situation, the characteristics of the thyroid nodule, and the recommended next steps. The goal is to gather more information about the nodule and determine the appropriate course of action based on your individual case. |
| Q2: Do I have cancer? | A Bethesda IV thyroid nodule result indicates that the cells obtained from the fine needle aspiration (FNA) biopsy show features suggestive of a follicular neoplasm, but the distinction between benign and malignant follicular neoplasms cannot be made definitively based on the FNA results alone. Therefore, having a Bethesda IV result does not confirm whether or not you have cancer.  The term "neoplasm" refers to an abnormal growth of cells, and follicular neoplasms can include both benign and potentially malignant conditions. Additional tests and evaluations are typically needed to determine the nature of the follicular neoplasm and whether it is cancerous.  Your healthcare provider may recommend further steps such as:  1. **Molecular Testing:** Molecular testing of the FNA sample may be considered to assess genetic or molecular markers associated with malignancy. This can provide additional information to help determine the risk of cancer.  2. **Thyroid Imaging:** Additional imaging studies, such as ultrasound or other diagnostic scans, may be used to evaluate the characteristics of the nodule and surrounding tissues.  3. **Surgical Consultation:** Due to the indeterminate nature of Bethesda IV results, your healthcare provider may refer you to a thyroid surgeon for a surgical consultation. In some cases, surgical removal of the nodule or a portion of the thyroid may be recommended for a definitive diagnosis.  It's important to discuss the specific findings and recommendations with your healthcare provider to understand your individual case and make informed decisions about further testing and treatment. The goal is to determine the nature of the thyroid nodule and develop an appropriate plan of action based on your unique circumstances. |
| Q3: What are my options? | If you have received a Bethesda IV thyroid nodule result (Follicular Neoplasm or Suspicious for a Follicular Neoplasm), further evaluation is typically needed to determine the nature of the follicular neoplasm and whether it is benign or malignant. Here are some common options:  1. **Molecular Testing:**  - Molecular testing of the fine needle aspiration (FNA) sample may be considered to assess genetic or molecular markers associated with malignancy. This can provide additional information to help determine the risk of cancer.  2. **Repeat FNA or Core Needle Biopsy:**  - Your healthcare provider may recommend a repeat FNA or a core needle biopsy to obtain a new sample for analysis. This can be done to gather more cellular material and improve the chances of making a definitive diagnosis.  3. **Thyroid Imaging:**  - Additional imaging studies, such as ultrasound or other diagnostic scans, may be performed to evaluate the characteristics of the nodule and surrounding tissues. Imaging can provide valuable information about the size, shape, and features of the nodule.  4. **Surgical Consultation:**  - Due to the indeterminate nature of Bethesda IV results, your healthcare provider may refer you to a thyroid surgeon for a surgical consultation. In some cases, surgical removal of the nodule or a portion of the thyroid (partial thyroidectomy) may be recommended for a definitive diagnosis.  5. **Clinical Observation:**  - In some cases, especially if the nodule is small and not causing significant symptoms, your healthcare provider may recommend a period of observation with regular monitoring through follow-up appointments and imaging studies.  The specific recommendation will depend on the individual characteristics of your case and your overall health. It's important to have a detailed discussion with your healthcare provider to understand the findings, the potential risks and benefits of each option, and the expected timeline for follow-up and further evaluation.  Thyroid nodules in the Bethesda IV category present a diagnostic challenge, and your healthcare team will work with you to determine the most appropriate course of action based on your unique circumstances. Regular communication with your healthcare team is crucial for making informed decisions about your care. |
| Q4: What is the best option for me? | When faced with a Bethesda IV thyroid nodule (Follicular Neoplasm or Suspicious for a Follicular Neoplasm), determining the best option for further evaluation and management depends on several factors, including your individual case, the characteristics of the nodule, and your overall health. Here are potential options that your healthcare provider may consider:  1. **Molecular Testing:**  - Molecular testing of the fine needle aspiration (FNA) sample may be recommended to assess genetic or molecular markers associated with malignancy. This can provide additional information to help determine the risk of cancer.  2. **Repeat FNA or Core Needle Biopsy:**  - A repeat FNA or a core needle biopsy may be recommended to obtain a new sample for analysis. This can be done to gather more cellular material and improve the chances of making a definitive diagnosis.  3. **Thyroid Imaging:**  - Additional imaging studies, such as ultrasound or other diagnostic scans, may be performed to evaluate the characteristics of the nodule and surrounding tissues. Imaging can provide valuable information about the size, shape, and features of the nodule.  4. **Surgical Consultation:**  - Due to the indeterminate nature of Bethesda IV results, your healthcare provider may refer you to a thyroid surgeon for a surgical consultation. In some cases, surgical removal of the nodule or a portion of the thyroid (partial thyroidectomy) may be recommended for a definitive diagnosis.  5. **Clinical Observation:**  - In certain cases, especially if the nodule is small and not causing significant symptoms, your healthcare provider may recommend a period of observation with regular monitoring through follow-up appointments and imaging studies.  The choice of the best option will be individualized based on your specific circumstances. It's crucial to have an open and detailed discussion with your healthcare provider to understand the findings, the potential risks and benefits of each option, and the expected timeline for follow-up and further evaluation.  Thyroid nodules in the Bethesda IV category present a diagnostic challenge, and your healthcare team will work with you to determine the most appropriate course of action based on your unique circumstances. Regular communication with your healthcare team is essential for making informed decisions about your care. |
| I have a Bethesda V thyroid nodule. | R17-20 |
| Q1: What does that mean? | A Bethesda V thyroid nodule refers to a specific category in the Bethesda System for Reporting Thyroid Cytopathology (TBSRTC). This system is used to classify the results of thyroid fine needle aspiration (FNA) biopsies, which are commonly performed to evaluate thyroid nodules. The Bethesda System categorizes the findings into six main diagnostic categories to guide further management, and Bethesda V is one of these categories.  Here's what Bethesda V means:  **Bethesda V - Suspicious for Malignancy:**  This category indicates that the cells obtained from the FNA biopsy are suggestive of malignancy. It implies a higher likelihood of cancerous cells being present in the thyroid nodule.  A Bethesda V result raises a significant concern about the potential for malignancy, and further diagnostic steps are typically recommended for a definitive diagnosis and to guide appropriate treatment.  Your healthcare provider may suggest additional tests or procedures, such as:  1. **Molecular Testing:** Molecular testing of the FNA sample may be considered to assess genetic or molecular markers associated with malignancy. This can provide additional information to help determine the risk of cancer.  2. **Thyroid Imaging:** Additional imaging studies, such as ultrasound or other diagnostic scans, may be performed to evaluate the characteristics of the nodule and assess any potential spread to nearby structures.  3. **Surgical Consultation:** Given the suspicion for malignancy, your healthcare provider may refer you to a thyroid surgeon for a surgical consultation. Surgical removal of the nodule or a portion of the thyroid (thyroidectomy) may be recommended for a definitive diagnosis and treatment.  It's essential to have a detailed discussion with your healthcare provider to understand the findings, the potential risks and benefits of each option, and the expected timeline for follow-up and further evaluation. A Bethesda V result indicates a significant concern, and prompt and comprehensive evaluation is typically recommended to determine the nature of the thyroid nodule and guide appropriate management. |
| Q2: Do I have cancer? | A Bethesda V thyroid nodule result (Suspicious for Malignancy) suggests a higher likelihood that the cells obtained from the fine needle aspiration (FNA) biopsy are indicative of malignancy, but it does not definitively confirm the presence of cancer. The Bethesda System for Reporting Thyroid Cytopathology is a standardized classification system that provides guidance on the likelihood of malignancy based on FNA results, but a definitive diagnosis often requires further evaluation.  To determine whether cancer is present and to guide appropriate treatment, your healthcare provider may recommend additional steps such as:  1. **Molecular Testing:** Molecular testing of the FNA sample may be considered to assess genetic or molecular markers associated with malignancy. This can provide additional information to help determine the risk of cancer.  2. **Thyroid Imaging:** Additional imaging studies, such as ultrasound or other diagnostic scans, may be performed to evaluate the characteristics of the nodule, assess any potential spread to nearby structures, and provide more information about the extent of the disease.  3. **Surgical Consultation:** Given the suspicion for malignancy, your healthcare provider may refer you to a thyroid surgeon for a surgical consultation. Surgical removal of the nodule or a portion of the thyroid (thyroidectomy) may be recommended for a definitive diagnosis and treatment.  It's important to discuss the specific findings and recommendations with your healthcare provider. They will be able to provide more information about the characteristics of the nodule, the potential risks and benefits of each option, and the expected timeline for follow-up and further evaluation.  A Bethesda V result is a significant concern, and prompt and comprehensive evaluation is typically recommended to determine the nature of the thyroid nodule and guide appropriate management. |
| Q3: What are my options? | If you have received a Bethesda V thyroid nodule result (Suspicious for Malignancy), further evaluation and management are typically warranted due to the increased likelihood of malignancy. Here are some common options that your healthcare provider may consider:  1. **Molecular Testing:**  - Molecular testing of the fine needle aspiration (FNA) sample may be recommended to assess genetic or molecular markers associated with malignancy. This can provide additional information to help determine the risk of cancer and guide treatment decisions.  2. **Thyroid Imaging:**  - Additional imaging studies, such as ultrasound or other diagnostic scans, may be performed to evaluate the characteristics of the nodule, assess any potential spread to nearby structures, and provide more information about the extent of the disease.  3. **Surgical Consultation:**  - Given the suspicion for malignancy, your healthcare provider may refer you to a thyroid surgeon for a surgical consultation. Surgical removal of the nodule or a portion of the thyroid (thyroidectomy) may be recommended for a definitive diagnosis and treatment.  4. **Whole-body Imaging (if necessary):**  - In some cases, additional imaging studies or scans may be recommended to evaluate whether the cancer has spread to other parts of the body. This is more common for aggressive types of thyroid cancer.  5. **Pathology Review:**  - Pathologists may conduct a detailed review of the FNA sample to confirm the findings and provide additional insights into the nature of the cells.  It's important to have a detailed discussion with your healthcare provider to understand the specific findings, the potential risks and benefits of each option, and the expected timeline for follow-up and further evaluation. The goal is to determine the nature of the thyroid nodule and guide appropriate management based on your individual circumstances.  A Bethesda V result raises a significant concern, and prompt and comprehensive evaluation is typically recommended to determine whether cancer is present and to guide appropriate treatment decisions. Regular communication with your healthcare team is essential for making informed decisions about your care. |
| Q4: What is the best option for me? | If you have received a Bethesda V thyroid nodule result (Suspicious for Malignancy), it's important to note that the best option for you will depend on various factors, including the specific characteristics of the nodule, your overall health, and your preferences. Here are potential options that your healthcare provider may consider:  1. **Molecular Testing:**  - Molecular testing of the fine needle aspiration (FNA) sample may be recommended to assess genetic or molecular markers associated with malignancy. This can provide additional information to help determine the risk of cancer and guide treatment decisions.  2. **Thyroid Imaging:**  - Additional imaging studies, such as ultrasound or other diagnostic scans, may be performed to evaluate the characteristics of the nodule, assess any potential spread to nearby structures, and provide more information about the extent of the disease.  3. **Surgical Consultation:**  - Given the suspicion for malignancy, your healthcare provider may refer you to a thyroid surgeon for a surgical consultation. Surgical removal of the nodule or a portion of the thyroid (thyroidectomy) may be recommended for a definitive diagnosis and treatment.  4. **Whole-body Imaging (if necessary):**  - In some cases, especially if there are concerns about potential spread, additional imaging studies or scans may be recommended to evaluate other parts of the body for cancer.  5. **Pathology Review:**  - Pathologists may conduct a detailed review of the FNA sample to confirm the findings and provide additional insights into the nature of the cells.  It's crucial to have an open and detailed discussion with your healthcare provider to understand the specific findings, the potential risks and benefits of each option, and the expected timeline for follow-up and further evaluation. The goal is to determine the nature of the thyroid nodule and guide appropriate management based on your individual circumstances.  A Bethesda V result raises a significant concern, and prompt and comprehensive evaluation is typically recommended to determine whether cancer is present and to guide appropriate treatment decisions. Regular communication with your healthcare team is essential for making informed decisions about your care. |
| I have a Bethesda VI thyroid nodule. | R21-24 |
| Q1: What does that mean? | A Bethesda VI thyroid nodule refers to a specific category in the Bethesda System for Reporting Thyroid Cytopathology (TBSRTC). This system is used to classify the results of thyroid fine needle aspiration (FNA) biopsies, which are commonly performed to evaluate thyroid nodules. Bethesda VI is the highest risk category and has a high suspicion for malignancy.  Here's what Bethesda VI means:  **Bethesda VI - Malignant:**  This category indicates that the cells obtained from the fine needle aspiration (FNA) biopsy are highly suspicious for malignancy. The likelihood of cancer is very high in Bethesda VI cases.  A Bethesda VI result strongly suggests the presence of cancer, and further steps for diagnosis and treatment are typically pursued promptly.  Your healthcare provider may recommend:  1. **Surgical Intervention:** Surgical removal of the thyroid nodule or a portion of the thyroid (thyroidectomy) is often recommended for a definitive diagnosis and to treat the suspected cancer.  2. **Whole-body Imaging (if necessary):** Additional imaging studies or scans may be performed to assess whether the cancer has spread to other parts of the body.  3. **Pathology Review:** Pathologists may conduct a detailed review of the FNA sample to confirm the findings and provide additional insights into the nature of the cells.  It's crucial to have an immediate and thorough discussion with your healthcare provider to understand the specific findings, the recommended course of action, and the expected timeline for treatment. A Bethesda VI result indicates a high suspicion for malignancy, and prompt and comprehensive evaluation is typically necessary to determine the extent of the cancer and guide appropriate treatment decisions. Regular communication with your healthcare team is essential for making informed decisions about your care. |
| Q2: Do I have cancer? | A Bethesda VI thyroid nodule result strongly suggests a high suspicion for malignancy. Bethesda VI is the category indicating that the cells obtained from the fine needle aspiration (FNA) biopsy are highly suspicious for cancer. While this category doesn't definitively confirm the presence of cancer, the likelihood of malignancy is very high.  If you have received a Bethesda VI result, your healthcare provider will typically recommend further steps for diagnosis and treatment, which may include:  1. **Surgical Intervention:** Surgical removal of the thyroid nodule or a portion of the thyroid (thyroidectomy) is often recommended for a definitive diagnosis and to treat the suspected cancer.  2. **Whole-body Imaging (if necessary):** Additional imaging studies or scans may be performed to assess whether the cancer has spread to other parts of the body.  3. **Pathology Review:** Pathologists may conduct a detailed review of the FNA sample to confirm the findings and provide additional insights into the nature of the cells.  It's important to have an immediate and thorough discussion with your healthcare provider to understand the specific findings, the recommended course of action, and the expected timeline for treatment. A Bethesda VI result raises a significant concern for malignancy, and prompt and comprehensive evaluation is typically necessary to determine the extent of the cancer and guide appropriate treatment decisions.  Keep in mind that every case is unique, and your healthcare team will tailor the approach to your specific situation. Regular communication with your healthcare providers is essential for making informed decisions about your care and understanding the next steps in the diagnostic and treatment process. |
| Q3: What are my options? | If you have received a Bethesda VI thyroid nodule result (Malignant), the recommendation for further management is typically focused on prompt and comprehensive treatment due to the high suspicion of malignancy. Here are common options that your healthcare provider may consider:  1. **Surgical Intervention:**  - Thyroidectomy: Surgical removal of the thyroid nodule or a portion of the thyroid is often recommended for both diagnosis and treatment. This allows pathologists to examine the tissue and confirm the presence of cancer. The extent of surgery may depend on factors such as the size and characteristics of the nodule.  2. **Lymph Node Dissection (if necessary):**  - If there is concern that cancer has spread to nearby lymph nodes, your surgeon may perform a lymph node dissection during the thyroidectomy to assess and remove affected lymph nodes.  3. **Postoperative Radioactive Iodine Treatment (if necessary):**  - After surgery, radioactive iodine therapy may be recommended to destroy any remaining thyroid tissue and microscopic cancer cells. This is especially common if the cancer is differentiated thyroid cancer (papillary or follicular).  4. **Thyroid Hormone Replacement Therapy:**  - After thyroid surgery, you may need thyroid hormone replacement therapy to replace the hormones that your thyroid would normally produce. This medication helps regulate your metabolism.  5. **Ongoing Follow-Up and Monitoring:**  - Regular follow-up appointments and monitoring are essential to assess your response to treatment, check for recurrence, and manage any potential side effects.  It's important to have a detailed discussion with your healthcare provider to understand the specific findings, the recommended course of action, and the expected timeline for treatment. Your healthcare team will work with you to tailor the approach based on the characteristics of the nodule, your overall health, and other individual factors.  Having a Bethesda VI result indicates a high suspicion of malignancy, and prompt and comprehensive evaluation and treatment are crucial for the best possible outcomes. Regular communication with your healthcare team is essential for making informed decisions about your care. |
| Q4: What is the best option for me? | If you have received a Bethesda VI thyroid nodule result (Malignant), the recommended course of action typically involves a comprehensive and timely approach to treatment. Here are key steps and options that your healthcare provider may consider:  1. **Surgical Intervention:**  - Thyroidectomy: Surgical removal of the thyroid nodule or a portion of the thyroid is often recommended for both diagnosis and treatment. This allows pathologists to examine the tissue and confirm the presence of cancer. The extent of surgery may depend on factors such as the size and characteristics of the nodule.  2. **Lymph Node Dissection (if necessary):**  - If there is concern that cancer has spread to nearby lymph nodes, your surgeon may perform a lymph node dissection during the thyroidectomy to assess and remove affected lymph nodes.  3. **Postoperative Radioactive Iodine Treatment (if necessary):**  - After surgery, radioactive iodine therapy may be recommended to destroy any remaining thyroid tissue and microscopic cancer cells. This is especially common if the cancer is differentiated thyroid cancer (papillary or follicular).  4. **Thyroid Hormone Replacement Therapy:**  - After thyroid surgery, you may need thyroid hormone replacement therapy to replace the hormones that your thyroid would normally produce. This medication helps regulate your metabolism.  5. **Ongoing Follow-Up and Monitoring:**  - Regular follow-up appointments and monitoring are essential to assess your response to treatment, check for recurrence, and manage any potential side effects.  The best option for you will depend on several factors, including the specific characteristics of the nodule, your overall health, and your preferences. It's crucial to have an open and detailed discussion with your healthcare provider to understand the specific findings, the recommended course of action, and the expected timeline for treatment. Your healthcare team will work with you to tailor the approach based on your individual circumstances.  Having a Bethesda VI result indicates a high suspicion of malignancy, and prompt and comprehensive evaluation and treatment are crucial for the best possible outcomes. Regular communication with your healthcare team is essential for making informed decisions about your care. |
| Overall | R1-24 |
